# Attainment of Community-Based Goals Is Associated with Lower Risk of Hospital Readmission for Older Australians Accessing the Australian Transition Care Program

**DOI:** 10.3390/ijerph22081162

**Published:** 2025-07-22

**Authors:** Salih A. Salih, Andrew Koo, Niamh Boland, Natasha Reid

**Affiliations:** 1 Centre for Health Services Research, The University of Queensland, Woolloongabba, Brisbane, QLD 4102, Australia; n.reid@uq.edu.au; 2 Redland Hospital, Queensland Health, Cleveland, QLD 4163, Australia; andrew.koo@health.qld.gov.au (A.K.); niamh.boland@health.qld.gov.au (N.B.); 3 Community and Oral Health Services, Metro South Hospital and Health Service, Cleveland, QLD 4163, Australia

**Keywords:** goal setting, transition care, behaviour change, older adults, quality of life

## Abstract

This study aimed to examine the 6-month hospital readmission rate for Transition Care Program (TCP) clients and its association with community goal attainment. This was a single-site retrospective cohort study of TCP clients admitted from 2014 to 2019. Goals were set at TCP entry and coded as goals ‘within the home’ or ‘in the community’. Hospital readmissions were tracked using electronic health records. Logistic regression, area under the curve, and number needed to treat were the primary analyses performed. Of 747 (66.8% female and 33.2% male) client episodes, 164 (22%) resulted in a hospital readmission. Clients who were not readmitted to hospital set and achieved a higher number of community-based goals (1.08 vs. 0.8, *p* = 0.01 and 0.8 vs. 0.6, *p* = 0.001). Utilising a logistic regression model, each additional community goal achieved was associated with a 30% reduction in risk of readmission to the hospital (OR: 0.69, 95%CI: 0.5–0.8; *p* = 0.002), adjusted for age, sex, MBI change, number of home goals achieved, hospital length of stay and number of comorbidities. Achieving community-based goals can reduce the risk of hospital readmission by 30% after adjusting for demographic and clinical variables.

## 1. Introduction

The Australian Transition Care Program (TCP) is a national service that provides short-term care and support to older adults who immediately post-discharge from hospital require additional assistance to regain their independence and improve their quality of life [1]. Eligibility for TCP is not dependent on pathology or diagnosis, but rather, individuals must be aged 65 or over (or 50 and over if they are of First Nations Descent), medically stable, and ready for discharge. Entry to the program requires a referral from a treating physician and assessment by an Aged Care Assessment Team Assessor to ensure that the individual is interested in this service, can articulate their rehabilitation goals, and will benefit from this period of restorative care [1]. TCP is a public health service delivered in a community-based or residential aged care facility-based setting, or a combination of both [2]. The cost of TCPs is subsidised by both state and federal governments in Australia, although recipients are expected to contribute a daily fee, which is calculated as a portion of their aged care pension [1]. TCPs are goal-oriented, slow-stream, and time-limited rehabilitation programs lasting up to 12 weeks with a possible extension for a further 6 weeks [1]. TCPs provide a range of services, including allied health services, nursing care, personal care, and domestic assistance. In a TCP, a care plan is developed by a Case Manager to meet the individual’s specific needs and goals. The number of people in TCPs has seen a yearly increase since its inception, from approximately 7000 to more than 25,000 people nationally at its peak. In 2017/18, there were 25,113 care recipients enrolled in the TCP, and the average age of these was 81 years, and 60 per cent were female [3]. Recipients received care for an average of 60 days [3].

A key objective of the TCP is to reduce hospital readmissions of older adults [4]. An initial evaluation of the program in 2008, shortly after its inception [5], found that, compared to a comparable group of older adults discharged from hospital without undergoing a TCP (n = 2188), those in a TCP (n = 2443) exhibited superior physical function, a reduced risk of hospital readmission, an extended time to readmission, and a diminished risk of entry to residential aged care within 6 months [6]. Despite significant investment in this program from government health departments, very few evaluations have occurred in the last 15 years, and it remains under-researched. Systematic reviews of similar transition care programs globally indicate their effectiveness in reducing the risk of unplanned hospital readmissions [4]. Given the costs and resources utilised in TCPs, understanding the role of Australian TCPs in the reduction in hospital readmissions across different client categories is imperative. The type of goals that clients set may be one such category to examine.

Goal setting is an important component of rehabilitation. It acts as a mechanism to align the objectives of clients with those of their rehabilitation team, ensuring that goals are personally meaningful to clients. By connecting care plans directly to the client’s goals [7], motivation is enhanced and the likelihood of behaviour change is increased [8]. Previous work by the research team has demonstrated that goals established by TCP clients align to the World Health Organization International Classification of Functioning Categories. Specifically, 74% of these goals can be categorised as home-based goals (activities within clients’ premises such as cooking and showering) and 26% as community-based goals (activities outside clients’ premises such as attending church, or visiting a friend) [9,10]. Additionally, this study also found that approximately 30% of clients set only home-based goals.

We hypothesise that TCP clients who establish community-based goals will exhibit a reduced risk of hospital readmissions compared to those who set home-based goals only. The attainment of community-based goals might be indicative of confidence in setting more diverse domestic and recreational goals coupled with the functional capacity to navigate the community [10]. This could contribute to enhanced levels of physical activity and increased social engagement, thereby potentially reducing the risk of hospital readmissions. Therefore, this study aimed to directly investigate whether the type of goals set by older adults, be they home-based or community-based, influences their likelihood of unplanned hospital readmission within 6 months.

## 2. Materials and Methods

### 2.1. Study Design

This was a single-site retrospective cohort study in a metropolitan health service that utilised data collected from a local database of the Transitional Care Program at Bayside Health (‘Transdata’) and the health service integrated electronic Medical Record (ieMR).

### 2.2. Setting

This project was conducted in one of three TCPs serving residents within the Metro South Health catchment area, Queensland, Australia. The population in the Redland Health catchment area in 2020 was approximately 160,331, with approximately 19% aged 65 years or older.

The program employed allied health professionals (occupational therapists, physiotherapists, speech pathologists, dietitians), allied health assistants, nurses, and personal care workers. Clinical staff members provided both discipline-specific care and case management services based on client needs. A supplementary brokerage model was utilised to ensure that TCP clients received the required service provided by allied health, nursing, and personal care as agreed in the individual care plan based on the clients’ needs [2]. The Bayside TCP site had a total of 50 TCP packages, 35 packages in the community and 15 packages in residential aged care facilities (RACFs). The average number of clients admitted to the program during the study period was 325 annually.

### 2.3. Participants

Clients discharged from hospital with a stable medical condition who were admitted to the Bayside TCP from 1 July 2014 to 31 December 2019 were included in this study. Clients also must have been discharged from the Bayside TCP to be included in the study; that is, they must not have been transferred to another TCP, re-admitted for a planned hospital admission and subsequently discharged without a TCP, or died during TCP admission. Only clients with complete data were included.

### 2.4. Dataset

The Bayside TCP use a local database, developed in 2010, called ‘Transdata’, that includes demographic and clinical variables. Goals statements, category, attainment and likelihood of attainment were recorded for each client at entry and program exit. Two researchers (NB and EM) completed data management together and reclassified goals as home-based or community-based goals. A third researcher (SS) was involved in the event of disagreement with goals classification to review and assist with decision-making.

#### Dataset Linking

Two researchers (NB and AK) used demographic data to link Transdata with ieMR using Unique Record Numbers (URNs). Unplanned hospital admission via a public hospital emergency department within six months of TCP discharge was obtained from ieMR. Planned hospital admissions, admissions for renal dialysis, admissions for elective surgery or day therapy, e.g., infusion, and emergency department presentations not requiring hospital admission were excluded. Clients with restricted access to their medical chart or monitored chart in ieMR were also excluded.

### 2.5. Measures

At TCP admission, demographic and clinical characteristics were obtained, including age, gender, living arrangement, length of hospital stay, reason for TCP admission, number of comorbidities, carer availability, hospital readmission while undergoing a TCP, and TCP discharge disposition and services. The length of TCP admission was also recorded. The Modified Barthel Index (MBI) was collected at entry and exit, we were goals and goal attainment, respectively. The goals’ ICF category.

#### 2.5.1. Modified Barthel Index

The MBI is a single measure of independence in activities of daily living (scale, 0 [severe functional dependence] to 100 [functional independence]) [11]. It is generally completed by the clients’ case manager at entry and discharge. The MBI has shown to have good inter-rater agreement with an intraclass correlation of 0.77 [12].

#### 2.5.2. Goals

At entry to a TCP, a collaborative discussion between the client, case manager and allied health staff to establish goals during the clients’ admission to the program was conducted. There was no standardised goal setting instrument used but rather SMART (Specific, Measurable, Attainable, Realistic, Timely) goals were applied and recorded in Transdata [13]. The type of goal (home vs. community), number of goals, and number of goals attained were also recorded. A summary of goals and their categorisation for a similar cohort is presented elsewhere [10].

Goals were categorised according to the International Classification of Functioning, Disability and health Framework (ICF) [9]. Community goals were defined as any intended action by the client outside their home boundary, whereas home goals were defined as any intended action by the client inside home boundaries. Based on this definition, ICF categorised goals can be classified as home or community goals. For example, a mobility goal of ‘to walk independently from the bedroom to the bathroom in two weeks’ is a home goal, while ‘to walk independently to the end of the road in four weeks’ is a community goal. Multidisciplinary team case conferences were held once weekly, led by a geriatrician, to discuss client progress and goal attainment. On discharge, goals were classified as ‘did not attain’, ‘partially attained’, or ‘fully attained’ [9].

### 2.6. Statistical Analysis

Goal attainment was categorised as attained and not attained, collapsing partially attained to attained. Continuous variable means were compared using t-tests and categorical variables were compared using chi-square tests; non-parametric tests were used for non-normally distributed data. Logistic regression was utilised to examine predictors of 6 months hospital readmissions (dependent variable). The independent variables included in the analysis were age, gender, entry MBI, MBI change, number of community goals achieved, number of home goals achieved, TCP length of stay, inpatient length of stay, client carer, and number of comorbidities. The variables with significant (*p* < 0.05) relationship with the outcome were included in the final logistic regression model. The Area Under the Curve (AUC) was calculated for the final model to determine predictive ability [14]. The Number Needed to Treat was calculated using the odds ratio and return to hospital event rate. All statistical analyses were performed at SPSS version 27.

## 3. Results

### 3.1. Client Characteristics

**Figure 1:**
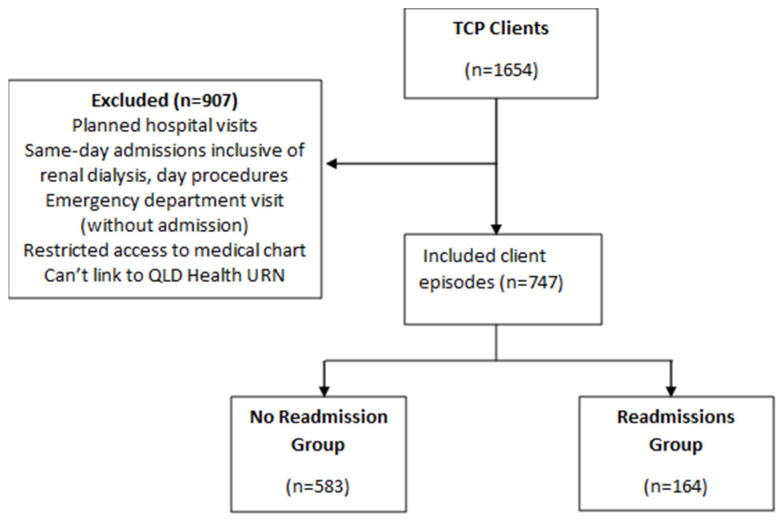
Patient Flowchart.

Out of 1654 TCP episodes, 747 client episodes were linked to Queensland health with Unique Record Numbers (URN). From these, 583 (78%) were not readmitted to hospital during the observation period, while 164 (22%) were readmitted to hospital (Figure 1).

In total, patients had a mean age of 80.3 years (SD = 8.1 years), predominantly female (66.8%), and the majority did not have a carer (74.1%). Analyses indicated significant differences between clients readmitted to hospital and those that were not. Readmitted clients were more likely to be male (40.2 vs. 31.2; *p* = 0.030), less likely to be discharged home with no additional services (16.5 vs. 24.7, *p* = 0.014) and have a carer (18.9 vs. 27.8, *p* = 0.022), and more likely to have been admitted to hospital for a medical, as opposed to surgical or orthopedic reason (51.8 vs. 39.5, *p* = 0.017). Readmitted clients also exhibited a lower exit MBI (74.2 vs. 75.9; *p* = 0.001), and a smaller change in MBI (11.5 vs. 14.4; *p* = 0.005) (Table 1).

### 3.2. Client Goals

Table 2 shows the differences in the number and type of goals set by clients eventually readmitted to hospital versus those that remained at home. Those readmitted set a lower number of overall goals (3.64 vs. 4.03; *p* = 0.006), achieved a fewer number of goals by discharge from the TCP (2.80 vs. 3.40; *p* < 0.001), including fewer community-based goals (0.60 vs. 0.83; *p* = 0.001).

### 3.3. Association Between Community-Goals and Readmission to Hospital

Table 3 shows the adjusted logistic regression analysis examining the association between the number of community goals achieved and hospital readmission. Adjusting for age, sex, change in MBI status, number of home goals achieved, carer status, and number of comorbidities, each additional community goals achieved was associated with a 30% reduction in risk of hospital readmission (OR = 0.699; 95% CI: 0.558, 0.875, *p* = 0.002). The ROC AUC for this model was 0.64; 95% CI 0.59 to 0.68, *p* < 0.001. Based on these results, the NNT was calculated to be n = 19.

## 4. Discussion

This study observed that, after adjusting for demographic and clinical variables, each additional community-based goal achieved was associated with a 30% reduction in the risk of hospital readmission. Home-based goal attainment, change in function or clinical variables were not associated with hospital readmission in adjusted models. This is the first study that investigates home versus community-based goals as a potential intervention mechanism.

One possible explanation for how setting and attaining community-based goals is associated with a lower risk of hospital readmission is through increases in incidental and planned physical activity. Time spent in hospital can result in hospital-acquired muscle deconditioning, leading to lower levels of physical activity and poorer outcomes [15]. A previous study led by this study’s lead researcher utilised an accelerometer to objectively assess activity patterns in TCP clients and found that they, on average, spend only 35 min walking each day [16]. This same study also found emerging evidence that a higher amount of walking time was associated with the achievement of more goals, although the association did not reach statistical significance.

Regular physical activity is strongly associated with better health outcomes across all ages [17]. There is a strong association between regular physical activity and reduced all-cause mortality, even if the physical activity is at a moderate level [18]. Moderate-intensity exercise for 30 min (or approx. 3000 additional steps [19]) on most days of the week lowers the risk of developing a range of chronic and life-threatening conditions, including dementia, obesity, cardiovascular disease, type 2 diabetes, stroke, dementia, and osteoporosis, as well as some cancers [20]. In addition, regular physical activity has been shown to improve both mood and cognitive function in older adults, and as such physical exercise is utilised in the management of chronic conditions such as depression, anxiety, chronic obstructive airway disease, arthritis, and chronic pain [21]. An increase of 30 min of physical activity on most days of the week also aligns with the Australian Physical Activity Guidelines for older adults [22].

Increasing physical activity, both incidental and planned, is crucial for older Australians participating in a TCP to reduce hospital readmissions. However, strategies to encourage older adults to increase their physical activity often prove to be ineffective. A recent systematic review and meta-analysis of physical activity programs for older people in the community receiving home care services found that adherence rates to the examined programs were low, mostly at 50% or less [23]. Identifying and setting personal goals for their rehabilitation journey may help older adults increase their incidental physical activity [24].

A second potential mechanism as to how attainment of community-based goals is associated with a lower risk of hospital readmission is through increases in the depth and frequency of social interactions. Social support, reduced loneliness or social isolation, and a sense of belonging are strong predictors of health and quality of life [25]. The risk of loneliness on mortality has been compared to that of obesity or smoking [26]. Furthermore, studies suggest a potential bi-directional relationship between social isolation and physical impairments, such that low physical function can lead to social isolation, and social isolation or loneliness can also result in poorer function [27]. Community-based goals, such as visiting friends, attending church, and generally participating in the community, may alleviate some of the negative consequences [28].

### Strengths and Limitations

Strengths of this study include a relatively large dataset and the use of a robust method via ICF classification for TCP goals to classify home versus community goals via two research team members and a third member in case of disagreement. However, the study was based on a single site in a metropolitan area of Brisbane, Australia, and findings may not be generalisable to other areas. The data was collected retrospectively, and the visibility of readmissions for clients outside of Queensland Health was limited, potentially missing clients who were readmitted to private or interstate hospitals. Another limitation of this study is its cross-sectional nature, meaning conclusions cannot be drawn about the causality of the identified relationships. While the analyses adjusted for baseline participant characteristics related to function and comorbidities, it remains plausible that more robust participants were the ones able to set and achieve community goals, rather than the community goals directly influencing outcomes. The data for nutrition and cognitive function was not uniformly collected throughout the study period and therefore not included in our models. Lastly, we did not investigate if there are differences in sub-types of community based goals (such as activity/mobility vs. social engagement), which is an area for future research. A further requirement for future research is to empirically test the causality of this relationship through a randomised controlled trial.

## 5. Conclusions

The study’s results reveal that for each community-based goal attained by an older adult in the Australian Transition Care Program, the likelihood of hospital readmission decreases by 30%. This suggests that establishing community-based goals may enhance the effectiveness of TCP participation. These findings reiterate the importance of underlying mechanisms associated with community participation, namely physical activity and social connectedness, to promote the achievement of key TCP objectives such as independence, companionship and quality of life [29,30].

Our preliminary findings might also provide a strategic direction to generate robust evidence regarding community goal setting based on the following: (i) the effectiveness of setting goals in achieving TCP objectives, (ii) adoption among older adults, (iii) implementation across TCPs with different socio-cultural demographics and (iv) maintenance over time in TCP care. Future randomised controlled trials should primarily be planned to understand the effectiveness of setting community-based goals (versus home-based goals) to achieve improved health-related outcomes supported by increased incidental physical activity and social connectedness among older adults. The assessment of setting community-based goals in terms of acceptability, ease of implementation and chances of maintenance in the long term is also needed to determine the feasibility of uniformly incorporating goal setting as a strategy in regular TCP care plans across Australia. Our findings may also be applicable beyond the Australian setting. The Australian TCP is delivered in a manner consistent with international examples found across 26 programs from 14 countries [31].

## Figures and Tables

**Table 1:** Differences in client demographics between those readmitted to hospital and those remaining at home.

Characteristics	Total N = 747 Episodes	No Readmission N = 583	Readmission N = 164	*p*-Value
Age; mean (SD)	80.3 (8.1)	80.4 (8.2)	80.03 (7.8)	0.560
Hospital Days LOS; mean (SD)	31.2 (28.9)	30.6 (29.7)	33.4 (26.2)	0.280
TCP Days LOS; mean (SD)	57.1 (27.2)	56.5 (26.5)	59.1 (29.5)	0.270
Entry MBI; mean (SD)	75.5 (15.7)	75.9 (15.4)	74.2 (16.9)	0.230
Exit MBI, mean (SD)	*89.4 (13.4)*	*90.5 (11.9)*	*85.7 (17.2)*	*0.001*
Change MBI, mean (SD)	*13.8 (11.6)*	*14.4 (11.8)*	*11.5 (10.6)*	*0.005*
Number comorbidities, mean (SD)	7.2 (2.8)	7.1 (2.8)	7.6 (2.8)	0.050
Male; n (%)	*248 (33.1)*	*182 (31.2)*	*66 (40.2)*	*0.030*
Discharge disposition, n (%)	*(N = 690)*	*(N = 533)*	*(N = 157)*	*0.014*
Home with no services	*158 (21.2)*	*132 (24.7)*	*26 (16.5)*	
Home with services	*496 (66.4)*	*379 (71.1)*	*117 (74.5)*	
RACF	*36 (4.8)*	*22 (4.1)*	*14 (8.9)*	
Client carer status, n (%)				0.022
Carer	193 (25.8)	162 (27.8)	31 (18.9)	
No carer	554 (74.1)	421 (72.2)	133 (81.1)	
Primary diagnosis category, n (%)				0.017
Medical	315 (42.2)	230 (39.5)	85 (51.8)	
General Surgery	45 (6.1)	38 (6.5)	7 (4.2)	
Orthopaedic	386 (51.7)	314 (53.9)	72 (44.0)	

LOS: Length of stay; MBI: Modified Barthel Index; RACF: Residential Aged Care Facility; SD: Standard Deviation. Italics indicates the statistically significant results.

**Table 2:** Differences in client goals between those readmitted to hospital and those remaining at home.

Goals	Total N = 747	No Readmission N = 583	Readmission N = 164	*p*-Value
At least one goal	741	579	162	0.300
*Number of goals; Mean (SD)*	*3.95 (1.6)*	*4.03 (1.6)*	*3.64 (1.7)*	*0.006*
*Total goals achieved; Mean (SD)*	*3.20 (1.7)*	*3.39 (1.7)*	*2.80 (1.7)*	*<0.001*
Number of home goals; Means (SD)	2.90 (1.5)	2.90 (1.5)	2.70 (1.4)	0.190
*Number home goals achieved; Mean (SD)*	*2.50 (1.5)*	*2.50 (1.5)*	*2.20 (1.6)*	*0.021*
*Number of community goals; Mean (SD)*	*1.03 (0.9)*	*1.08 (0.9)*	*0.80 (0.9)*	*0.010*
*Number of community goals achieved; Mean (SD)*	*0.78 (0.8)*	*0.83 (0.8)*	*0.60 (0.7)*	*0.001*

Italics indicates the statistically significant results.

**Table 3:** Adjusted logistic regression analysis of hospital readmission risk.

Independent Variable	Odds Ratio	95%CI	*p* Value
Number of community goals achieved	0.70	0.56 to 0.88	0.002
Age on admission	0.99	0.97 to 1.01	0.342
Female	0.71	0.49 to 1.02	0.063
MBI Change	0.98	0.97 to 1.00	0.051
Number of home goals achieved	1.05	0.99 to 1.25	0.064
Has carer	0.68	0.43 to 1.05	0.082
Number of comorbidities	1.06	0.99 to 1.13	0.064

## Data Availability

The data presented in this study are available on request from the corresponding author due to privacy reasons.

## Abbreviations

The following abbreviations are used in this manuscript:

AUC	Area Under the Curve
ICF	International Classification of Functioning
ieMR	Integrated electronic Medical Record
LOS	Length of Stay
MBI	Modified Barthel Index
OR	Odds Ratio
RACF	Residential Aged Care Facility
TCP	Transition Care Program
URN	Unique Record Number
